# Catastrophic Antiphospholipid Syndrome in a Young Female Complicated by Systemic Lupus Erythematosus and Left Atrial Myxoma: A Rare Case Report

**DOI:** 10.1002/ccr3.72933

**Published:** 2026-06-14

**Authors:** Nazmin Ahmed, Mohammad Nazrul Hossain, Abdullah Salman, Sabrina Yesmin, S. M. G. Saklayen

**Affiliations:** ^1^ Department of Neurosurgery Ibrahim Cardiac Hospital & Research Institute Dhaka Bangladesh; ^2^ Department of Rheumatology BIRDEM Geeral Hospital Dhaka Bangladesh; ^3^ Department of Vascular Surgery Ibrahim Cardiac Hospital & Research Institute Dhaka Bangladesh

**Keywords:** catastrophic antiphospholipid syndrome, myxoma, systemic lupus erythematosus, thrombosis

## Abstract

Catastrophic antiphospholipid syndrome (CAPS) is a fulminant and rare variant of antiphospholipid syndrome characterized by rapidly progressive multiorgan thrombosis and a high mortality rate. Its diagnosis is often challenging due to overlapping clinical features with sepsis, thromboembolic disorders, and systemic autoimmune diseases. We report a rare and complex case of CAPS in a young woman with newly diagnosed systemic lupus erythematosus, further complicated by a left atrial myxoma, which resulted in widespread arterial thrombosis, acute ischemic stroke, pulmonary involvement, and peripheral vascular occlusion. Early clinical suspicion, prompt immunomodulatory therapy, anticoagulation, and coordinated multidisciplinary care resulted in significant clinical and radiological improvement. This case highlights the importance of early diagnosis and aggressive treatment in CAPS, particularly when unusual thrombotic triggers coexist.

## Key Clinical Message

Concomitant occurrence of catastrophic antiphospholipid syndrome, systemic lupus eryrhematosus and cardiac myxoma is one of the rarest but life‐threatening condition requiring rapid recognition and aggressive multidisciplinary management to improve outcomes.

**Abbreviations**
APS: antiphospholipid syndrome
CAPS: catastrophic antiphospholipid syndrome
CT: computed tomography
IVIG: intravenous immunoglobulin
LA: lupus anticoagulant
LMWH: low molecular weight heparin
MCA: middle cerebral artery
MRI: magnetic resonance imaging
SLE: systemic lupus erythematosus

## 1 Introduction

Antiphospholipid syndrome (APS) is an autoimmune disorder defined by recurrent arterial, venous, or microvascular thrombosis and/or obstetric morbidity occurring in association with the persistent presence of lupus anticoagulant (LA), anticardiolipin, and/or anti‐β2‐glycoprotein in samples taken 12 weeks apart [1]. Catastrophic Antiphospholipid syndrome (CAPS) is a rare and severe form of APS characterized by multiple vascular occlusive events (≥ 3 events), typically in small‐vessel, occurring over a short period (≤ 7 days), with histopathologic confirmation and persistent anti phospholipid antibodies over ≥ 12 weeks [1]. Systemic lupus erythematosus (SLE) is the most common autoimmune condition associated with secondary APS [1]. Cardiac tumors such as atrial myxomas are rare but are recognized as causes of systemic embolization. However, the coexistence of CAPS, SLE, and atrial myxoma is exceptionally uncommon and poses a significant challenge from diagnosis to management. Herein, we report a young female who presented with this complex diagnosis and no prior history of any manifestation of these diseases.

## 2 Case Presentation

### 2.1 Case History and Examination

A 26‐year‐old woman presented with a 3‐day history of fever, acute onset right‐sided weakness, slurred speech and shortness of breath. She had no prior history of OCP use or other medication use, spontaneous abortion, or similar illness in first‐degree relatives. General examination demonstrated an ill looking patient with average body build and nutrition. Her BMI was 22. Peripheral pulses were absent in all four limbs except for carotid pulses, and blood pressure was non‐recordable. All extremities were cold without overt gangrene. Besides this, a few scattered ulcerations were noted inside the oral cavity. There were no palpable lymph nodes and the condition of the skin was normal. Neurological examination demonstrated her Glasgow Coma Scale score 14/15, right‐sided hemiparesis (MRC: 0/5) with an extensor plantar response. Respiratory system examination demonstrated tachypnea, diminished breath sounds with widespread fine crepitation in both lung fields.

### 2.2 Differential Diagnosis

As the patient was a young female presented with features of stroke, non‐recordable blood pressure and pulse in absence of shock, our provisional diagnosis was that of Takayasu's arteritis. Important differentials to be considered were autoimmune disease, like‐SLE, Antiphospholipid antibody syndrome.

### 2.3 Investigations

Table 1 summarizes the Biochemical, Hematological and Immunological findings of the patient. Laboratory investigations revealed Moderate Microcytic Hypochromic Anemia (Hb 7.7 g/dL), elevated inflammatory markers, raised cardiac biomarkers, elevated D‐dimer levels, Serum ANA strongly positive and a moderately positive LA. However, other auto antibody panel, like ENAs, dsDNA, anti‐phospholipid IgA, and anti‐cardiolipin IgA, M, or G showed negative result. Beside this, patient had the features of sepsis with positive blood and urine culture for *Candida parapsilosis* and *Klebsiella pneumoniae* respectively.

**TABLE 1:** Biochemical analysis of the patient supporting the diagnosis.

Investigation	Value (after initial admission)	Value (after administration of IVIG)	Value (re‐admission after 3 months)	Reference value	Remarks
CBC	Hb: 7.7 g/dL, PCV: 25.2%, WBC: 6630/mm^3^, N: 88%, L: 9%, PLT: 230,000/μL	Hb: 11.9 g/dL, PCV: 37.1%, WBC: 7660/mm^3^, N: 80%, L: 13%, PLT: 201,000/μL	Hb: 10.6 g/dL, PCV: 32.2%, WBC: 6700/mm^3^, N: 82%, L: 12%, PLT: 132,000/μL	Hb: 11.6–15 g/dL, WBC: 4000–11,000/mm^3^, N: 50%–70%, L: 20%–40%, PLT: 150,000–300,000/μL	Thrombocytopenia as additive diagnostic criteria for SLE
S. Procalcitonin	0.58 ng/mL	—	—	< 0.10: Normal 0.10–0.50: Local or systemic bacterial infection can be possible > 0.50 to < 2.0: progress to sepsis > 2.0 to < 10.0: sepsis	Progress to sepsis, which act as triggering factor for CAPS
CRP	6.8 mg/L	2.10 mg/L	16.5 mg/L	< 6 mg/L	Inconclusive, administration of steroid can alter the result
PT, INR	14.1 s, 1.01	14.5 s, 1.05	32.1 s, 2.68	PT: 12–15 s	Therapeutic anticoagulation ↑ value
S. Creatinine	1.01 mg/dL	0.52 mg/dL	1.0 mg/dL	0.6–1.4 mg/dL	Within normal limit
D‐Dimer	0.95 μg/mL	0.48 μg/mL	—	0.00–0.50 μg/mL	Inconclusive
HBsAg, Anti HCV, Anti HIV	Negative	—	—	—	Negative
Troponin‐I, hs	588.6 pg/mL	Not done	—	At 99th percentile: Male: 76.2 Female: 51.4	Hypertroponinemia, probably due to sepsis
NT Pro BNP	6470 pg/mL	735 pg/mL	—	Cut off value 125 for < 75 years Cut off value 450 for ≤ 75 years	Early sign of heart failure
Urine R/E	Sp. Gravity: 1.00, RBC: 20–25/HPF, EC: A few, PC: 1–2/HPF, cast: nil	Sp. Gravity: 1.008, RBC: Nil, EC: 6–8/HPF, PC: 0–2/HPF, cast: nil	Sp. Gravity: 1.011, RBC: 21–40/HPF, EC: 0–3/HPF, PC: 3–5/HPF, cast: nil, Bacteria: +++	—	New onset hematuria signifies microvascular thrombosis in CAPS
ANA	Immunofluorescence observed. Anti nuclear antibody strongly positive. Pattern: cytoplasmic antibody	—	—	—	Fulfills entry criteria for diagnosis of SLE, according to 2019 EULAR/ACR criteria
LAs	LA1: 49.1 s LA2: 30.8 s LA1/LA2 Ratio: 1.59	LA1: 44.2 s LA2: 35.8 s LA1/LA2 Ratio: 1.23	LA1: 117.2 s LA2: 53.3 s LA1/LA2 Ratio: 2.20	> 2: strongly positive 1.5–2: moderately positive 1.2–1.5: weakly positive	Repeat LA after 3 months showed strong positivity
Anti‐phospholipid Ab (IgG)	3.1 GPL‐U/mL	3.2 GPL‐U/mL	3.4 GPL‐U/mL	< 10 GPL‐U/mL: negative > 10 GPL‐U/mL: positive	Negative
Anti‐phospholipid Ab (IgM)	5.1 MPL‐U/mL	3.4 MPL‐U/mL	4.5 MPL‐U/mL	< 10 MPL‐U/mL: negative > 10 MPL‐U/mL: positive	Negative
P‐ANCA	1.5 U/mL	—	—	< 5 U/mL: negative > 5 U/mL: positive	Negative
C‐ANCA	1.7 U/mL	—	—	< 5 U/mL: negative > 5 U/mL: positive	Negative
Anti‐Cardiolipin Antibody, IgG	3.1 GPLU/mL	3.1 GPLU/mL	2.8 GPLU/mL	< 10 GPLU/mL: negative > 10 GPLU/mL: positive	Negative
Serum IgG (nephelometry)	2066.0 mg/dL	—	—	700–1600 mg/dL	Positive
Serum IgA (nephelometry)	99.30 mg/dL	—	—	70–400 mg/dL	Negative
Serum IgM (nephelometry)	184.0 mg/dL	—	—	40–230 mg/dL	Negative
Anti‐Thyroglobulin Ab	1.3 IU/ML	—	—	< 4.5 IU/mL negative ≥ 4.5 IU/mL positive	Negative
Anti‐Thyroid peroxidase Ab	< 37.0 U/mL	—	—	< 60 U/mL negative > 60 IU/mL positive	Negative
Urine C/S with MIC	Growth of *Klebsiella pneumoniae*	—	Growth of *Klebsiella spp*	—	Positive
Blood C/S‐Fan method with MIC	Growth of *Candida parapsilosis*	—	Negative	—	Positive
Fungus (1–3) beta D Glucan Ag	> 1200.0 pg/mL	—	—	< 80: negative 80–100: indeterminate > 100: positive	Positive
Anti Thrombin III	100.60%	—	—	75.00%–126.00%	Within normal limit
Protein C	99.20%	—	—	70.00%–140.00%	Within normal limit
Protein S	94.40%	—	—	70.00%–125.00%	Within normal limit

Abbreviations: IVIG, intravenous immunoglobulin; LA, lupus anticoagulant.

**FIGURE 1:**
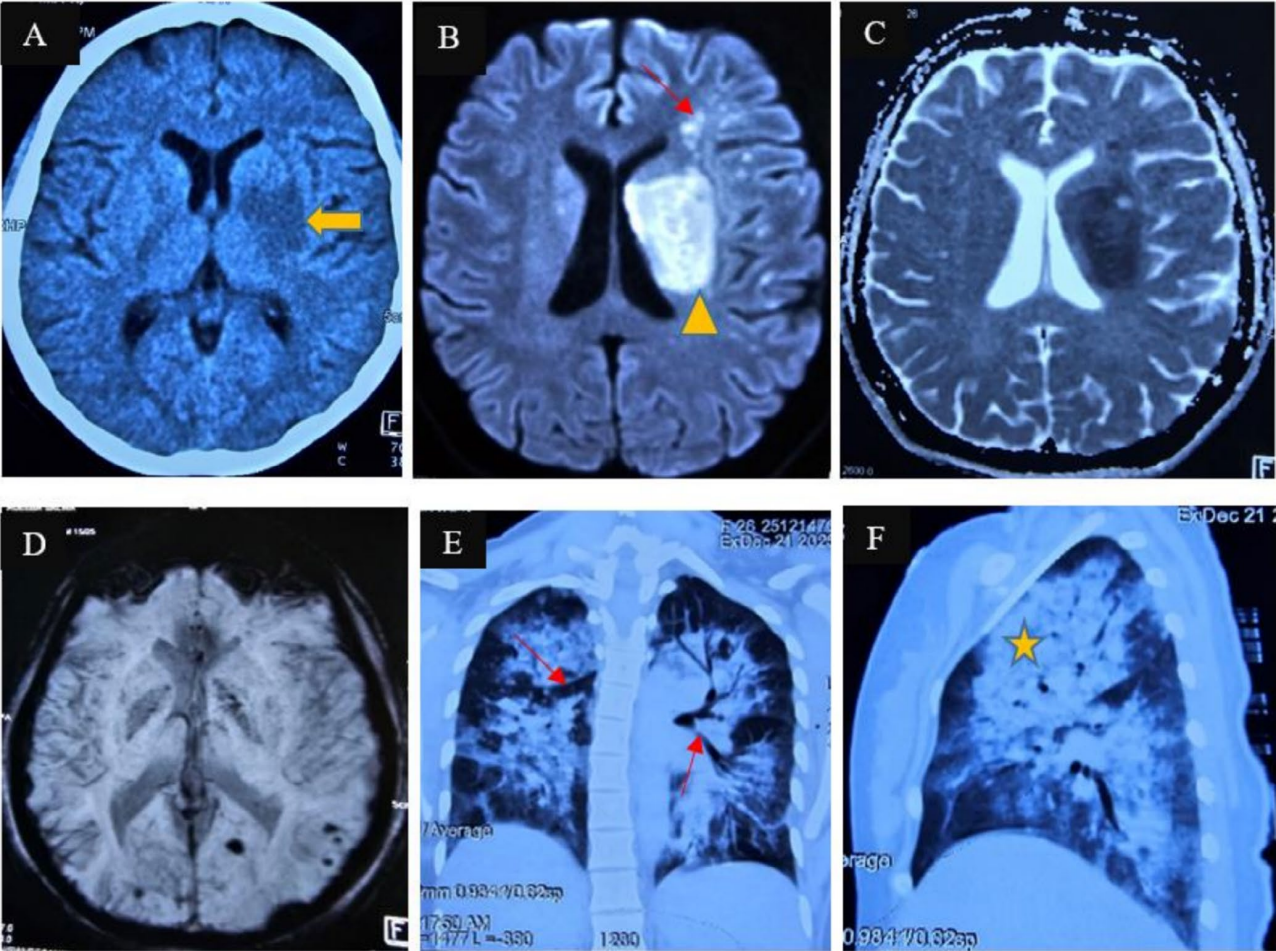
CT scan of the brain (A) demonstrated left‐sided capsule‐ganglionic ischemia, following MCA territory (marked by orange arrow), which showed restricted diffusion in DWI/ADC mapping (B and C, marked by orange arrowhead). Besides this, small scattered multiple areas of restricted diffusion also noted in left fronto‐parietal area (marked by red arrow). The area showed no haemorrhagic transformation in SWI sequence (D). Moreover, HRCT of the chest demonstrated diffuse alveolar hemorrhage with intervening ‘dark bronchus sign’ (E, marked by red arrow) and widespread consolidation (F, marked by orange star). ADC, apparent diffusion coefficient; CT, computed tomography; DWI, diffusion‐weighted imaging; MCA, middle cerebral artery; SWI, susceptibility weighted image.

Computed tomography (CT) scan of the brain demonstrated left‐sided capsulo‐ganglionic ischemia in the middle cerebral artery (MCA) territory. This infarct was more clearly shown by the diffusion restriction on diffusion‐weighted imaging/apparent diffusion coefficient images. Additional small scattered areas of restricted diffusion were seen in the left fronto‐parietal region, with no evidence of hemorrhagic transformation on susceptibility weighted image images of magnetic resonance imaging (MRI). HRCT of the chest revealed diffuse alveolar hemorrhage with a “dark bronchus sign” and widespread consolidation (Figure 1).

**FIGURE 2:**
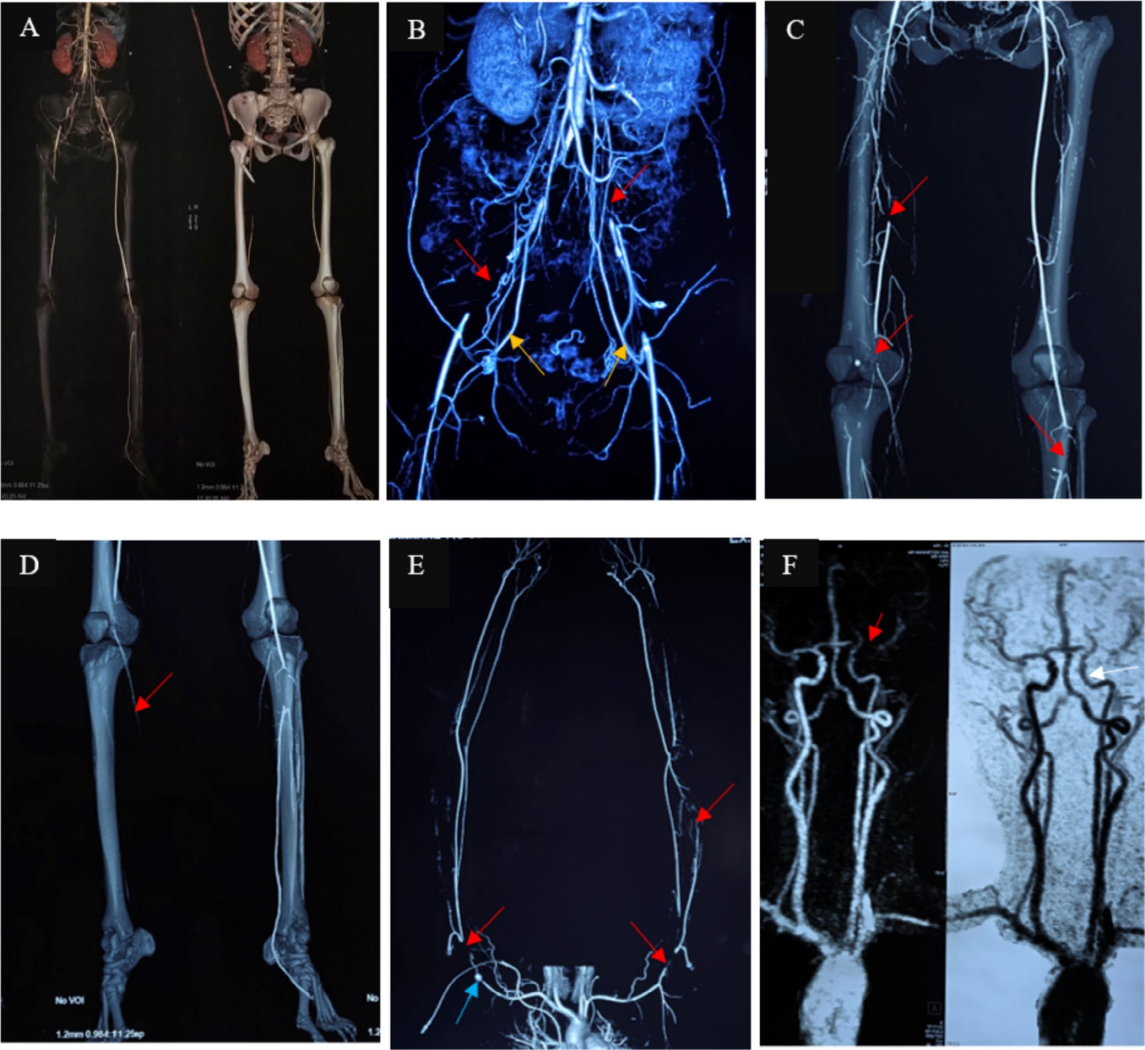
CT angiogram of abdominal aorta and lower limb demonstrated extensive arterial thrombosis, involving proximal left external and internal iliac arteries, mid and distal right common iliac artery with extension into right external and internal iliac arteries (A, B), proximal right mid and lower superficial femoral artery, occlusion of left popliteal artery (C), non‐visualization of distal part of right anterior tibial, posterior tibial and peroneal arteries (D). CT Angiogram of Upper limb arteries demonstrated aneurysm of right mid axillary artery, followed by total occlusion. Besides this, total occlusion of left mid/distal axillary artery, osteo‐proximal brachial artery and bilateral interosseous arteries were also noted (E). MR Angiogram of cerebral vessel showed narrowing of left ICA with non‐visualization of M1 segment of left MCA (F). CT, computed tomography; MCA, middle cerebral artery.

Due to the absence of all peripheral pulses, we did CT angiography of both upper and lower limbs which showed extensive arterial thrombosis in the abdominal aorta and lower limbs, involving the iliac arteries, right superficial femoral artery, and occlusion of the left popliteal artery, with non‐visualization of distal leg arteries. CT angiography of the upper limbs revealed a right axillary artery aneurysm with complete occlusion and occlusion of the left axillary, proximal brachial, and bilateral interosseous arteries. MR angiography showed narrowing of the left internal carotid artery with non‐visualization of the M1 segment of the left MCA (Figure 2).

**FIGURE 3:**
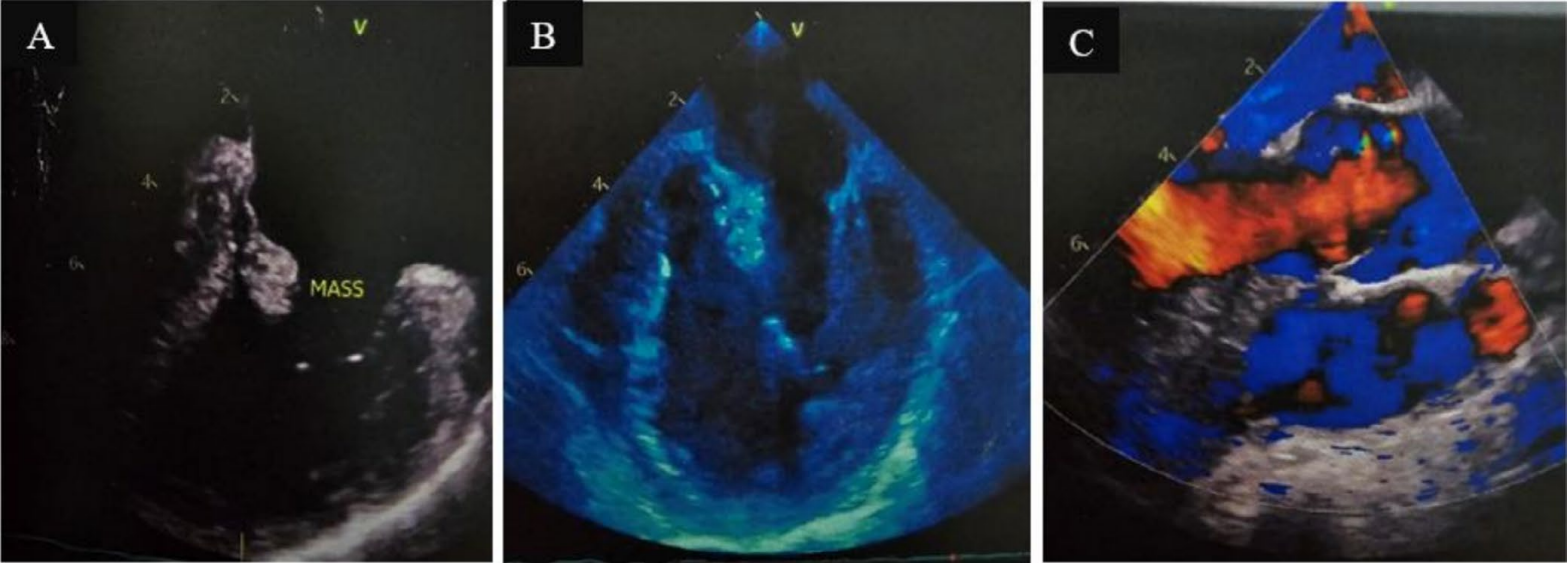
Photograph of trans‐esophageal echocardiogram demonstrated a pedunculated mass attached to the near insertion point of AML into the left ventricle during diastole, suggestive of LA myxoma. There were no vegetation/thrombus (A, B). Eccentric moderate mitral regurgitation was noted (C). LA, lupus anticoagulant.

Besides this, trans‐esophageal echocardiography identified a pedunculated multilobulated mass attached to the anterior mitral leaflet, consistent with a left atrial myxoma, along with inferior vena cava thrombosis. The surface of the mass was irregular and it moved during diastole (Figure 3).

Based on the rapid multiorgan involvement, extensive arterial and venous thrombosis, positive antiphospholipid antibodies and ANA, fulfilling both entry and additive criteria of SLE, a diagnosis of CAPS secondary to SLE, complicated by left atrial myxoma, was established.

### 2.4 Management

A multidisciplinary team involving cardiology, rheumatology, neurology, vascular surgery, cardiac surgery, and intensive care specialists opted for conservative management. According to the decision of medical board, the patient received intravenous methylprednisolone 500 mg 12 hourly for 3 days, followed by gradual tapering within next 10 days, therapeutic anticoagulation with low molecular weight heparin (LMWH), enoxaparin 60 mg 12 hourly in subcutaneous route and Intravenous IgG (0.4 g/kg/day) for 5 sessions. Her biochemical and hematological markers favored the presence of both bacterial and fungal infection (Table 1), hence she was also given intravenous vancomycin and meropenem for 14 days and intravenous voriconazole for the same duration.

Over the following days, the patient showed marked neurological improvement. Her other systemic parameters also stabilized. A repeat magnetic resonance angiography (MRA) was done that showed improved peripheral blood flow in the upper and lower limbs. However, the peripheral pulses were still weak. Subsequently, various immunological tests were done, including Serum Anti‐Nuclear Antibody that showed a positive result in Indirect Immunofluorescence assay. This fulfilled the entry criteria of SLE, according to 2019 EULAR/ACR criteria for diagnosis of SLE.

Initially, plasmapheresis was prescribed as a part of the treatment. But the patient deferred it due to financial constraints. She was discharged on oral anticoagulant (apixaban), oral antiplatelet, and oral corticosteroids in tapering dose with close outpatient follow‐up. After clinical stabilization, she was planned for elective surgical excision of the atrial myxoma with mitral valve intervention after 3 months.

### 2.5 Follow Up

**FIGURE 4:**
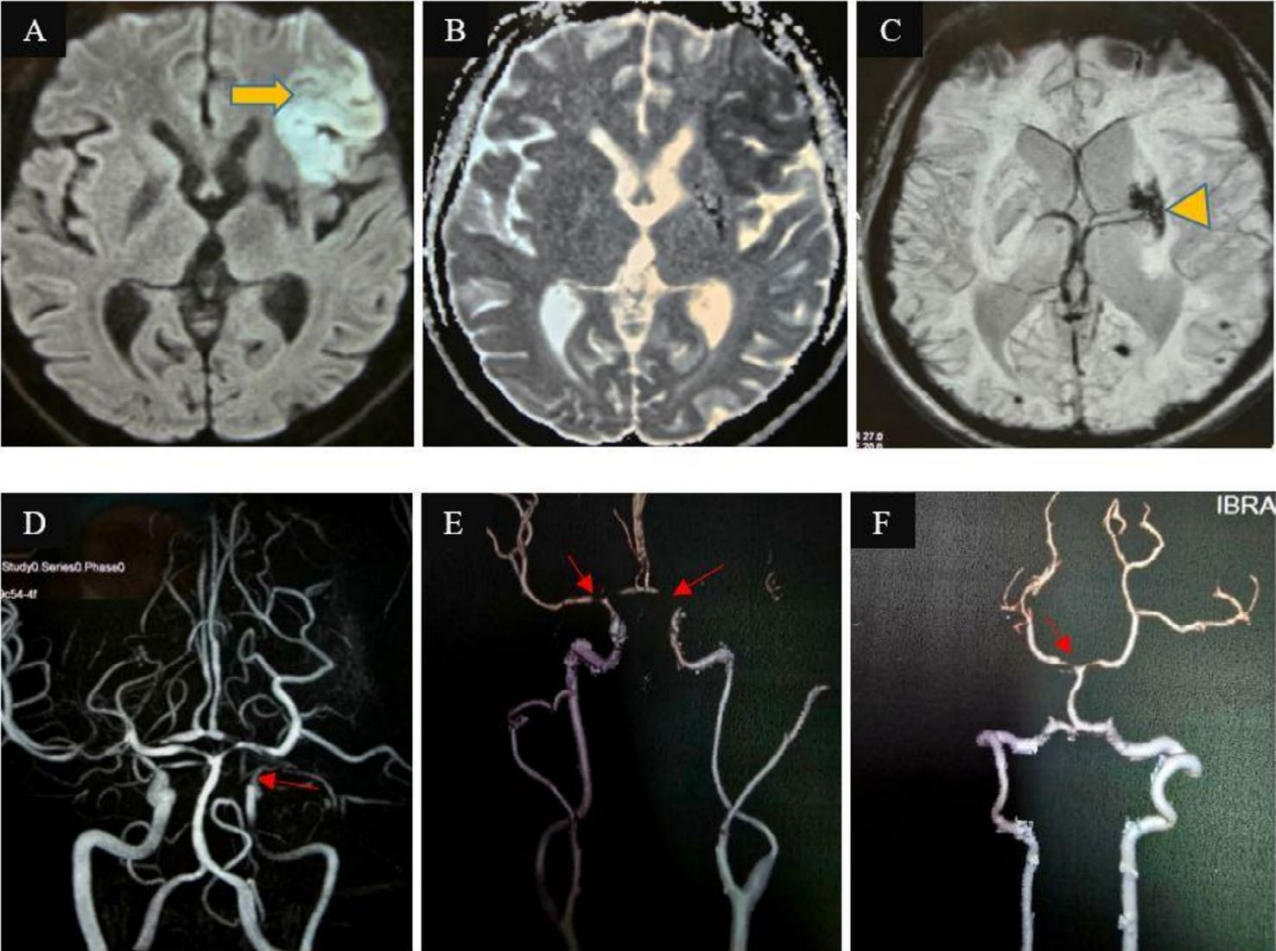
Follow up MRI of Brain, after 3 months demonstrated an area of restricted diffusion in left frontal lobe (A and B, marked by yellow arrow). Additionally, haemorrhagic transformation noted in left capsule‐ganglionic area (C, marked by yellow arrowhead). Both MRA (D) and CTA (E, F) demonstrated diffuse moderate to severe disease of left ICA, ipsilateral occlusion of M1/M2, A2 segment, right P1 and M1 segment (marked by red arrow). MRI, magnetic resonance imaging.

The patient was on regular follow up under the department of Rheumatology. After 1 month, the patient suddenly developed hepatitis and was managed conservatively at another hospital. Due to repeated infections 1 month apart, a steroid‐sparing agent could not be started. At 3 months follow up, her muscle power improved (MRC‐3/5 in right upper limb and 2/5 in lower limb). However, she suddenly developed features of aphasia. Repeat MRI of brain after readmission demonstrated a new onset ischemia involving left frontal lobe with features of hemorrhagic transformation in left basal ganglia. Besides this, both MRA and CTA demonstrated diffuse moderate to severe disease of left ICA, ipsilateral occlusion of M1/M2, A2 segment, right P1 and M1 segment (Figure 4). Besides this, repeat biochemical and serological markers showed persistent LA and presence of UTI (Table 1). The patient was treated conservatively and she was planned for intravenous immunoglobulin (IVIG) again after subsidence of infection. Aphasia improved and muscle power remained the same as at admission.

## 3 Discussion

CAPS is a rare but devastating manifestation of antiphospholipid syndrome, occurring in almost 1% of all reported APS cases [1]. Despite optimal treatment, it still carries a high risk (37%–48%) of mortality, that increases significantly if it is associated with SLE [1, 2].

This particular case is extremely rare due to the coexistence of CAPS, SLE and a left Atrial myxoma. All three together resulted in a rapidly progressive, widespread arterial and venous thrombosis [3]. Although CAPS is most associated with SLE, cardiac myxoma has only been reported sporadically as a precipitating factor [3]. Each of these entities can act both as a source of emboli and a trigger for endothelial activation and cytokine release [4, 5].

The pathophysiology of CAPS remains partially understood to date. As implied by the “triple‐hit” hypothesis, additional pro‐thrombotic factors should be present to transform a state of hypercoagulability into CAPS. Nearly 60% of patients appear to have one or more triggering factors, especially infections (30%–50% of cases), pregnancy (22%), surgical interventions or trauma (17%), neoplasms (16%), contraceptives (10%), or the discontinuation of anticoagulation. Underlying mutations in complement regulatory genes could serve as a “second hit,” leading to uncontrolled complement activation and a more severe thrombotic phenotype. This “double” or “triple‐hit” hypothesis may apply to any patient with multi‐organ failure [1, 2, 3, 4, 5, 6]. Concomitant presence of both bacterial and fungal infection with the presence of sepsis (Table 1) in our reported case supports this hypothesis. Moreover, the presence of pedunculated myxoma, surface irregularity, and attachment with the anterior mitral leaflet with its movement during the diastolic phase further contributed to the turbulent flow and leads to this widespread thrombosis.

The diagnosis of CAPS is often difficult because its clinical presentation closely resembles several other life‐threatening conditions, including sepsis, disseminated intravascular coagulation, heparin‐induced thrombocytopenia, and various thrombotic microangiopathies such as thrombotic thrombocytopenic purpura, hemolytic uremic syndrome, and HELLP syndrome (hemolytic anemia, elevated liver enzymes, and low platelet count) [6]. In this patient, early fever, pulmonary infiltrates, cerebral infarct, thrombosis in the limb vessels, and elevated inflammatory markers initially obscured the proper diagnosis. Additionally, autoimmune serology was initially inconclusive. This emphasizes the importance of maintaining a high suspicion when rapid multi‐organ thrombosis occurs [7].

Radiologically, CAPS typically involves small‐ and medium‐vessel thrombosis; however, this case demonstrated extensive large, medium, and small vessel occlusion involving cerebral, aortoiliac, and peripheral arteries. Such diffuse large to small artery involvement is distinctly uncommon and can mimic vasculitis or primary embolic disease [7]. Pulmonary consolidation further complicated differentiation from infection or acute respiratory distress syndrome, both recognized mimickers of CAPS [8]. CAPS is primarily characterized by diffuse thrombotic microangiopathy with a predilection for the kidneys, lungs, brain, heart, skin, and gastrointestinal tract [1, 8]. Specifically, the clinical presentation of CAPS is characterized by kidney involvement in 73% of cases, with varying degrees of kidney dysfunction, and pulmonary involvement in 60% of cases, presenting as acute respiratory distress syndrome or pulmonary embolisms (26%) [9]. Up to 56% of patients exhibit cerebral manifestations, such as stroke or encephalopathy [10]. The heart is affected in half of cases, primarily as myocardial infarction or valvulopathy. Libman–Sacks endocarditis is reported in 13% of CAPS with cardiac involvement [9, 10]. However, the 2023 ACR/EULAR APS classification is likely to contribute to the revision of CAPS classification criteria, providing a better understanding of APS and its various clinical and biological phenotypes [1, 9, 10]. According to this criteria, our reported case presented with simultaneous involvement of cardiovascular, respiratory, and nervous system, less than a week with laboratory confirmation of persistent LA antibody in > 12 weeks apart (Table 1). In presence of anticoagulant therapy, detection of LA may demonstrate false positive and false negative result [11]. As our reported patient was on LMWH, there is less chance of a false positive result. Though we had no evidence of histopathological confirmation of small vessel occlusion, still the presentation and laboratory criteria fulfill the diagnosis of probable CAPS.

The patient's clinical course was further complicated by the concurrent diagnosis of SLE. Serum Anti‐Nuclear antibody that showed a positive result in Indirect Immunofluorescence assay fulfills the entry criteria of SLE according to 2019 EULAR/ACR criteria for diagnosis of SLE [12]. Moreover, history of fever (2 points), delirium (2 points), oral ulcer (2 points), thrombocytopenia (4 points), LA (2 points) fulfills the additive criteria.

The currently available treatment guideline of CAPS recommends urgent anticoagulation along with aggressive immunomodulation [1, 9]. The combination of anticoagulation, high‐dose corticosteroids and IVIG improved the chances of survival. In CAPS associated with SLE, cyclophosphamide may be beneficial [10]. Despite the inability to perform plasma exchange in this case, early initiation of anticoagulation, corticosteroids, and IVIG resulted in significant clinical improvement. On the other hand, broad spectrum antibiotics and antifungal medication played a pivotal role to control infection, resulting in rapid clinical stabilization of the patient. Surgical excision of the atrial myxoma was halted until patient stabilization from CAPS, aligning with published recommendations [13].

## 4 Conclusion

CAPS is a medical emergency requiring high clinical suspicion, especially in young patients with SLE and unexplained widespread thrombosis. Rare concomitant conditions such as atrial myxoma may act as triggering factors. Prompt multidisciplinary management can significantly improve survival and functional outcomes.

## 5 Learning Points

- CAPS should be suspected in young patients with rapid‐onset multiorgan thrombosis, even when initial autoimmune tests are negative.
- Extensive large‐vessel arterial occlusion does not exclude CAPS and may represent an atypical radiologic manifestation.
- Rare triggers such as cardiac myxoma can precipitate CAPS by promoting embolization and inflammatory activation.
- Early multidisciplinary management and prompt immunomodulatory therapy are critical for improving outcomes in CAPS.

## Author Contributions

**Nazmin Ahmed:** conceptualization, data curation, formal analysis, investigation, writing – original draft. **Mohammad Nazrul Hossain:** supervision, validation, visualization, writing – review and editing. **Abdullah Salman:** data curation, investigation, writing – review and editing. **Sabrina Yesmin:** supervision, validation, writing – review and editing. **S. M. G. Saklayen:** conceptualization, investigation, supervision.

## Funding

The authors have nothing to report.

## Consent

Written informed consent was taken from the patient prior to initiation of this project.

## Data Availability

Data available on request from the corresponding author.
